# Neural Responses to Infant Emotions and Emotional Self-Awareness in Mothers and Fathers during Pregnancy

**DOI:** 10.3390/ijerph17093314

**Published:** 2020-05-09

**Authors:** Cristina Trentini, Marco Pagani, Marco Lauriola, Renata Tambelli

**Affiliations:** 1 Department of Dynamic and Clinical Psychology, “Sapienza” University of Rome, 00185 Rome, Italy; renata.tambelli@uniroma1.it; 2 Institute of Cognitive Sciences and Technologies, CNR, 00185 Rome, Italy; marco.pagani@istc.cnr.it; 3 Department of Social and Developmental Psychology, “Sapienza” University of Rome, 00185 Rome, Italy; marco.lauriola@uniroma1.it

**Keywords:** pregnancy, mothers and fathers, emotional self-awareness, infant emotions, hdEEG

## Abstract

Neuroscientific research has largely investigated the neurobiological correlates of maternal and (to a much lesser extent) paternal responsiveness in the post-partum period. In contrast, much less is known about the neural processing of infant emotions during pregnancy. Twenty mothers and 19 fathers were recruited independently during the third trimester of pregnancy. High-density electroencephalography (hdEEG) was recorded while expectant parents passively viewed images representing distressed, ambiguous, happy, and neutral faces of unknown infants. Correlational analyses were performed to detect a link between neural responses to infant facial expressions and emotional self-awareness. In response to infant emotions, mothers and fathers showed similar cerebral activity in regions involved in high-order socio-affective processes. Mothers and fathers also showed different brain activity in premotor regions implicated in high-order motor control, in occipital regions involved in visuo-spatial information processing and visual mental imagery, as well as in inferior parietal regions involved in attention allocation. Low emotional self-awareness negatively correlated with activity in parietal regions subserving empathy in mothers, while it positively correlated with activity in temporal and occipital areas implicated in mentalizing and visual mental imagery in fathers. This study may enlarge knowledge on the neural response to infant emotions during pregnancy.

## 1. Introduction

“Infant survival and development depend on communication with a caregiver to service the baby’s needs for an emotional *attachment*, but also to maintain and develop an intimate emotionally expressed *companionship* in changing purposes and conscious experiences” [1] (p. 7).

From birth, infants are precociously able to engage with others and share their subjective states. Such early abilities, that have been extensively described in the theory of “innate intersubjectivity” (Trevarthen, 1998), are sustained by parents’ propensity to recognize, empathize with, and respond to the emotional signals of their infants [2,3]. Investigations employing audio-visual microanalysis of mother–infant interactions have provided evidence that mothers rapidly modify their behavior in response to the emotional cues of their children [4] to develop mutual coordination based on emotional sharing [2,5]. These parental behavioral adjustments to infant emotions have been defined as intuitive (that is, faster than controlled conscious responses), since they occur within a temporal interval of 200–800 ms [6]. Mother’s readiness for parenting is reflected by a repertoire of behaviors—including gaze, smile, ‘‘motherese’’ vocalizations, and affectionate touch [7,8]—which have been shown to facilitate communicative skills [9] and attachment security in children [10].

Sensitive and attuned parental behaviors are activated by the peculiar features of the infant’s physiognomy (which is characterized by big eyes, a high and protruding forehead, chubby cheeks, small nose, mouth, and chin), and plump body shape [11,12]. Such features constitute a “baby schema”—or, as postulated by Lorenz [13,14]—, a *Kindchenschema*, which represents a biologically relevant stimulus to which adults, especially parents, are highly motivated to respond [13,14,15,16,17,18]. *Kindchenschema* is conceptualized as an “innate releasing mechanism” for caregiving behavior and emotional orientation towards infants [13]. Such an evolutionary perspective is in line with Seligman’s theory of preparedness, which postulates the ability (observed also in animal species) to respond automatically to cues—particularly those expressing threats—that are critical for survival [19]. Behavioral studies have provided evidence of this preparedness, documenting that humans respond earlier to infants’ emotional signals, as compared to adult faces and other social stimuli [16,20,21]. 

Considering the high relevance of emotional infant signals, behavioral studies have largely explored the adaptive function of preparedness in parents. Recent neuroimaging investigations have found that, for mothers, infant faces preferentially engage attention compared to adult faces [22]. Studies employing electroencephalography (EEG) have provided further support to these findings, showing that mothers exhibit event-related potential (ERP) that reflect greater attention allocation to their own child’s face compared to the faces of unfamiliar children or adults [23]. ERP studies have also shown that, compared to non-mothers, mothers report greater neural activity in response to infant facial expressions. Moreover, contrary to what has been observed in non-parents, parents’ neural responses are influenced by the degree of infant distress [24]. Behavioral research has provided evidence that proneness to respond to infant distress is well established in women during late pregnancy. Indeed, expectant mothers take longer to disengage attention from distressed infant faces compared to happy infant faces, showing an attentional bias to infant distress which, in turn, is significantly related to enhanced mother–infant bonding during the postpartum period [25]. These findings suggest that the ability to recognize infant emotions during pregnancy significantly affects mothers’ ability to respond sensitively to their infants’ emotional cues after they are born.

### 1.1. Parental Sensitivity, Affect Regulation, and Emotional Self-Awareness

Parental sensitivity relies on the ability to accurately perceive and interpret the emotional underpinnings of the infant’s overt behavior and to respond appropriately to them [26]. These parental competences are strictly affected by the complex reorganization processes that occur during pregnancy to allow both mothers and fathers to acquire a parental identity and to develop an emotional bond with their still unborn infant [27,28,29,30,31,32]. 

Parents’ proneness to interpret infant inner states provides an essential scaffolding for the development of communication competencies [9], attachment security, and socio-emotional abilities in the infant [33,34,35]. It is noteworthy that mothers and fathers differ in the way they express sensitivity during interactive exchanges with their children. Maternal sensitivity is mainly manifested through tenderness and emotional support and is aimed at regulating a child’s aversive arousal in times of distress or needs [12,36]. On the other hand, paternal sensitivity is manifested through vigorous playful stimulations and is aimed at encouraging the child to joyfully open up to the outside world, while setting proper limits for his/her safety [37,38,39,40]. Thus, even with different purposes, mothers and fathers act as “external regulators” for the child [41]. 

Parental successful affect regulation is conceived as the ability to maintain an inner balanced emotional state in the face of the need to modulate a child’s arousal and—at the same time—to promote the growth of his/her regulatory strategies [42]. As Barrett et al. have evidenced, for these regulatory strategies to be used, parents need prior awareness of their emotional states [43].

A multi-dimensional subclinical condition in which emotional self-awareness is compromised or severely disrupted is alexithymia [44,45]. Alexithymia is a stable personality trait [46], characterized by difficulties recognizing and describing emotions in oneself as well as by the tendency to minimize emotional experience by focusing on external events [47]. While the first two dimensions of alexithymia describe deficits in emotional self-awareness, the third factor refers to the tendency to avoid emotional thinking [48,49]. Emotional self-awareness has been conceptualized as an attentional process that, beyond sustaining the monitoring, the differentiation, and the description of emotions, enables an individual to understand the reasons behind each emotional state and to recognize its physiological correlations [50].

As Bagby and Taylor have underlined, individuals who have poor awareness of their own emotional states “cannot readily imagine themselves in another person’s situation and are consequently unempathetic and ineffective in modulating the emotional states of others’’ [51] (pp. 30–31). Consistent with such assumptions, several investigations have evidenced that individuals with alexithymic profiles have an impaired ability to identify emotions from facial expressions. Interestingly, these deficits, beyond including negative emotions (such as sadness, anger, and fear), also concern happiness and surprise [44,52,53].

While research has extensively studied the effects of alexithymia on parenting during the postpartum period, the relationship between alexithymia and parental responses to infant emotional cues during pregnancy has been overlooked in scientific literature. A large number of studies have provided evidence that alexithymia impairs maternal ability to recognize, interpret, empathize with, and regulate the emotional states of the child [54,55,56,57]. Although there is still a paucity of investigations on fathers, existing data show that men who have difficulties identifying and describing feelings are also prone to be emotionally distant from their children [58]. These relational experiences may impede the development of a secure attachment in the child [59], obstructing the developmental trajectories of those brain areas that subserve the ability to explore, communicate, and regulate emotions [53,60]. Given that alexithymia is a stable personality trait [46], it might be expected that this condition could also negatively affect parental response to infant emotions during pregnancy.

### 1.2. Research on Neural Correlates of Parental Responsiveness to Infant Cues

The fundamental changes that occur during pregnancy and the post-partum period sustain parental sensitivity and reactivity to infant visual, auditory, and olfactory cues [61,62,63,64]. In women, these behavioral changes are modulated by the large amounts of hormones secreted during pregnancy, and (later) during birth, lactation, and physical contact with their baby [63,65,66]. Among these hormones, vasopressin and oxytocin have a crucial role in stimulating bonds between mothers and infants [67]. Interestingly, during pregnancy and in the early postpartum weeks, hormonal transformations also occur in men’s brains (even if this is to a lesser degree than in mothers), showing biochemical profiles that are similar to those of women [68,69]. 

Over the past years, a large body of research employing neuroimaging techniques has investigated the neurobiological correlates of parental responsiveness to infant cues, including baby cries and infant emotional faces: most of these investigations have been focused on mothers, while few have investigated fathers [70,71].

Data coming from neuroimaging studies indicate that maternal brain responses to infant emotional signals are modulated by the connections between highly conserved and automatic brain circuitry (which are similar to those underpinning parental care in rodents), and later-evolving paralimbic and cortical structures implicated in higher-order socio-affective processes, such as empathy, mentalizing, and emotion regulation [71,72,73,74,75]. The existing (although still scarce) literature on fathers shows that infant emotional faces increase activity in several cerebral regions (prefrontal, striatal, and insular areas) that overlap with those revealed in mothers [74]. 

The parental subcortical-limbic network includes the amygdala, the hypothalamus, and the dopaminergic reward circuit, considering (for the latter) both its mesolimbic (that is, nucleus accumbens and ventral tegmental area) and nigrostriatal (that is, striatum and substantia nigra) pathways [74,76,77]. The medial preoptic area of the hypothalamus (MPOA) and the hippocampus play a significant role in maternal behaviors, with the former sustaining the initiation of maternal behavior and the latter modulating memory and learning. In a pioneering structural brain study, Kim et al. found an increase of gray matter volume in the maternal midbrain (including the hypothalamus and substantia nigra) and amygdala, which was correlated with a maternal positive perception of the infant, thus evidencing a link between neurobiological plasticity and parental affective representations [78]. These structures are strictly connected to paralimbic and cortical networks that are implicated in empathy, emotion regulation, and mentalizing.

The cortical network underpinning empathy (best known as the mirror neuron system; MNS) includes the inferior frontal gyrus (IFG), the supplementary motor area (SMA), the inferior and superior parietal lobule (IPL and SPL), and the superior temporal sulcus (STS) [79,80,81,82]. SPL provides sensorimotor contributions to the MNS, through which actions, emotions, and sensations observed in the others can be collapsed into the observer’s neurobiological substrate, enabling empathy, as documented in previous studies on mothers [80]. The insula is a detection center for arousal and salience of emotional stimuli [83]. This structure serves as a relay between the frontal components of the MNS (which provide a motor representation of the observed or imitated facial expression) and the limbic system (which is instead involved in emotion processing) [84]. In this manner, motor representations of others’ facial expressions are translated into their emotional significance, which can, in turn, be simulated (that is, automatically experienced) [79]. These cerebral processes are associated with the activation of SMA, a premotor area connected to the precuneus, which is implicated in the high-order aspects of motor control, such as planning and selection of movements [85]. In mothers, activity in the pre-SMA has been interpreted as a promptness to act, aimed at providing the infant with caring behaviors [80]. 

Cortical mentalizing network—which comprises the superior temporal sulcus/gyrus (STS/STG), the precuneus, the posterior cingulate cortex (PCC), the temporal parietal junction (TPJ), and the ventromedial prefrontal cortex (vmPFC) [86,87,88,89]—allows parents to infer and interpret infant mental states (e.g., intentions, motivations, and feelings) [74]. Previous research has found that, among areas included in vmPFC, medial frontal gyrus (MFG) specifically activates when mothers are confronted with ambiguous infant expressions, thus reflecting the cognitive efforts mothers make to ascribe a significance to these uncertain emotions [80].

These processes are fundamental in enabling parents to respond to infant cues sensitively, through the activation of the emotion regulation and executive network, which includes the frontopolar cortex (FPC), the dorsolateral prefrontal cortex (DLPFC), the orbitofrontal cortex (OFC), and the medial frontal gyrus (MFG) [90,91]. Among these areas, OFC has great relevance in parenting, since it is involved in socio-emotional behaviors and affect regulation processes underlying the attachment bonds [27,90,92]. OFC (particularly its medial part; mOFC) also has a crucial role in modulating adults’ affective propensity towards infants, since it responds after only 130 ms from the presentation of an infant face [18]. Based on this evidence, it has been suggested that mOFC is “a potential brain basis for the ‘innate releasing mechanisms’ described by Lorenz for affection and nurturing of young infants” [18] (p. 5). 

Finally, a further significant region in parenting is the anterior cingulate cortex (ACC). ACC is activated during reward-based decision making [93,94]. Moreover, because of its projections to both the “emotional” limbic system (i.e., nucleus accumbens) and the “cognitive” prefrontal cortex (PFC), ACC has a key role in integrating the neuronal circuitry involved in emotional awareness and affects regulation [95].

### 1.3. Study Aims

Neuroscientific research is enlarging the knowledge of the neural processing of infant emotions in mothers and (to a much lesser extent) in fathers, during the postpartum period, when the parental brain is progressively modeled by parent–child interactions and, thus, by the experience of parenthood itself. In contrast, much less is known about the cerebral areas implicated in the response to infant emotional cues during pregnancy, when both mothers and fathers are still facing the psychological reorganization as well as the neurobiological processes that will allow them to detect and sensitively respond to their infants’ emotions after childbirth. Moreover, while the consequences of emotional self-awareness on parenting have been largely documented in the postpartum period, the effects of emotional self-awareness on the response to infant emotions in expectant parents has yet to be explored. 

Starting from these premises, we used high-density electroencephalography (hdEEG) to investigate the neural response to infant emotions during late pregnancy in mothers and fathers, respectively. In line with previous neuroimaging studies [80,81], we investigated this issue by comparing single emotions (that is, distressed, happy, and ambiguous) to neutral facial expressions. We also tested the correlations among the neural responses to infant facial expressions and the levels of emotional self-awareness, in mothers and fathers respectively.

Consistent with neuroscientific literature on parents, we expected that, in response to infant distressed and happy emotions, mothers and fathers will show similar enhanced activity in cerebral areas implicated in empathy, mentalizing, and emotion regulation processes. Nevertheless, given the evidence of a specific maternal inclination to detect and respond to infant negative emotions, we also expected that, when confronted with infant distress, only mothers will show enhanced activity in premotor areas implicated in motor planning and motor control (SMA). We hypothesized that these neural responses would be indicative of an early “promptness to act” [80], oriented to prepare mothers to reassure the infant.

Moreover, we expected that infant ambiguous emotions will mainly elicit a cerebral signal increase in regions implicated in mentalizing processes (specifically, STS/STG, PCC, TPJ, and MFG), both in mothers and in fathers. We hypothesized that these neural responses would reflect the efforts that both mothers and fathers made to ascribe an emotional meaning to these uncertain facial expressions [80].

Lastly, given the crucial role played by emotional self-awareness on the ability to identify emotions from facial expressions [44,52] and on the quality of parental behavior [54], we expected that, both in mothers and in fathers, low emotional self-awareness will be associated with decreased activity in cerebral areas, subserving parental ability to infer, empathize with, and regulate infants’ emotions.

As we are aware of no investigation exploring the relationship between emotional self-awareness and neural response to infant emotions among mothers and fathers during late pregnancy, we believe that our study may contribute to enlarging the knowledge on the processes underlying the transition to parenthood.

## 2. Materials and Methods 

### 2.1. Participants and Procedures

Initially, we recruited 31 primiparous mothers and 29 primiparous fathers from maternity and child health services, during the third trimester of pregnancy. These subjects took part in a larger extensive investigation, whose aim was assessing the effects of early interventions on parents at risk of psychopathological symptoms and on their children’s emotion regulation abilities during the first year of life. The data presented in this paper concern the first step of this broader study, which took place during pregnancy, when we screened mothers and fathers for anxious and depressive risk conditions. Our original intention was to enroll parents coming from the same families with no anxious and depressive symptomatology. Because the screening of parental psychological characteristics did not reveal any family in which both parents were within the normal range, we had to select parents coming from different families. 

Among the eligible parents, 1 mother and 3 fathers dropped out of the study, thus, the final sample constituted 20 mothers aged between 23 and 44 years (*M* = 34.85; ± 5.06), and 19 fathers aged between 24 and 49 years (*M* = 37.21; ± 6.61). All parents were right-handed, married or cohabitating with their partners, and came from middle socioeconomic status backgrounds. 

Before data collection, parents received complete information about the study procedures and handed over their written informed consent to participate in the research, in accordance with the Declaration of Helsinki. This study was approved by the Ethics Committee of the authors’ Institution (Prot. n. 0,001,002 - July 15, 2019 - UOR: SI000092 - Classif. VII/15).

### 2.2. Psychopathological Screening

The State-Trait Anxiety Inventory Y form (STAI-Y) [96,97] and the Edinburgh Postnatal Depression Scale (EPDS) [98,99] were administered to detect and exclude anxious and depressive symptoms in both mothers and fathers.

The STAI-Y is a self-report scale designed to measure both state (STAI-S) and trait (STAI-T) anxiety. State and trait subscales include 20 items with a four-point Likert scale, ranging from 1 to 4. Scores higher than 41 indicate clinically significant symptoms for both state and trait anxiety. In mothers, the mean scores for STAI-S and STAI-T were 29.50 (±4.14; range 23–37) and 27.90 (±4.61; range 20–35), respectively. In fathers, the mean scores for STAI-S and STAI-T were 28.79 (±2.91, range 20–33) and 28.58 (±2.71, range 23–33), respectively.

The EPDS is a 10-item self-report questionnaire used to screen for depressive symptoms during pregnancy and in the post-partum period. Each item is scored on a 4-point scale (ranging from 0 to 3) and rates the intensity of depressive symptoms during the previous 7 days. Scores higher than 9 indicate “possible depression” both in mothers [99] and in fathers [100,101]. The mean score for EPDS was 1.4 (±.68; range 0–2) in mothers and 1.05 (±.71, range 0–2) in fathers, respectively.

### 2.3. Psychological Measures: Emotional Self-Awareness

Subjects completed the 20-item Toronto Alexithymia Scale [102,103]. The TAS-20 is one of the most widely used self-report measures of alexithymia, a multi-dimensional subclinical condition which reflects low levels of emotional self-awareness, since it is characterized by a difficulty identifying and describing feelings, and difficulties distinguishing feelings from body sensations along with a tendency to minimize emotional experience by focusing on external events. Respondents are asked to rate 20 items on a 5-point Likert scale, ranging from 1 to 5. The total score is obtained by summing the scores on the following three factors: difficulty identifying feelings (TAS-20/F1); difficulty describing feelings (TAS-20/F2); and externally oriented thinking (TAS-20/F3). Higher scores on TAS-20/Tot identify subjects with possible (52≤ x ≤ 60), or certain alexithymia (x ≥ 61). To explore the possible link between the single components of emotional self-awareness and neural responses to infant facial emotions, only the three TAS-20 subscales were considered for correlational analyses.

### 2.4. hdEEG Stimuli

**Figure 1:**
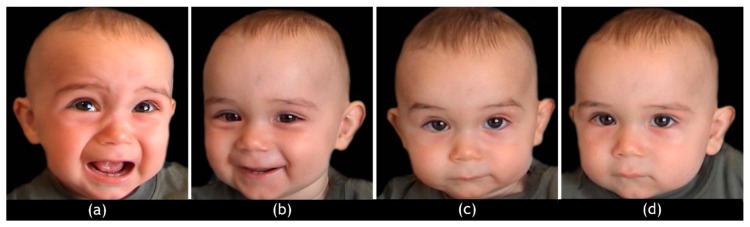
Example of a set of infant facial expressions shown to the parents. (Distress (**a**); Happy emotions (**b**); Ambiguous emotions (**c**); Neutral expressions (**d**)).

At each hdEEG recording, distressed (D), happy (H), ambiguous (A), and neutral faces (N) (24 of each) were presented full–screen on a 15” color monitor, in randomized and unpredictable order (Figure 1).

The pictures represented colored frontal headshots of six children (three females and three males, aged between 6 and 12 months) selected from previous studies [80,81,104], with every expression being represented by four pictures of each child.

Infant facial expressions were identified according to precise criteria. “D” emotions are defined by brows drawn together and lowered to create a mid-brow bulge, a deepened naso-labial furrow and tight squeezing of the eye orbit muscles resulting in a strong squint, and a widened mouth with corners lowered [105]; “H” emotions are characterized by narrowed eyes, an arched eyebrow, and a widened mouth with corners raised; “A” emotions are characterized by blended expressions and the co-presence of different facial mimic patterns both in the upper and lower areas of the face; finally, “N” faces are defined by brows raised slightly, wide-open eyes, relaxed mouth with semi-opened or (rarely) closed lips, and none naso-labial folds [106,107]. The pictures were uniform in brightness, shading, and size of the head. Each picture was presented twice for 1500 ms, with an inter-trial interval (ITI) of 1000 ms. Parents were instructed to simply look at the pictures and pay attention to the infants’ faces.

### 2.5. hdEEG Recordings

Stimuli presentation was controlled through a PC running E-Prime Software, Version 2.0 (E–Prime^®^ 2.0). A two hundred and fifty-six channel dense array EEG was recorded using the Geodesic Sensor Net (Electrical Geodesics Inc. Eugene, OR). The electrodes net montage required approximately 15 min and was well tolerated by the parents. Data were digitized at 512 Hz. All channels were referenced to the vertex electrode (Cz). Impedance was kept below 10 kΩ. 

### 2.6. Data Analyses

#### 2.6.1. Psychological Measures

A series of two-sample T-tests were performed to assess between-group differences in TAS-20 sub-scales (F1, F2, and F3) and total (TAS-20-Tot) scores.

#### 2.6.2. hdEEG Processing and Analysis

hdEEG raw files were exported in binary format and converted by Statistical Parametric Mapping 12 (SPM12) into “mat files.” The latter were coregistered to the virtual template corresponding to the hdEEG headset. Based on published literature, epoching was performed for each trial on the temporal window 100–400 ms and the resulting files high–pass filtered at 0.1Hz. Artifacts were removed by a flat–segment method; the resulting files underwent robust averaging. The analyzed range of the frequency bands was 0–30 Hz.

Source analysis was performed by a prior space modeling that (in the absence of an individual structural scan) allowed the creation of individual head meshes describing the boundaries of different head portions, based on each subject’s structural scan. These meshes were generated by applying the inverse of the spatial deformation field that maps the individual structural image to canonical meshes derived from the Montreal Neurological Institute (MNI) template. Subsequent steps of source analysis included: data coregistration to the MNI template; forward computation according to the boundary element method (BEM); inverse reconstruction by applying multiple sparse priors (MSP) algorithm; and the creation of a three-dimensional Neuroimaging Informatics Technology Initiative (3D NIfTI) image [108].

For each subject, NIfTI images were generated by 96 trials for each experimental condition (D, H, A, and N). The flexible factorial routine of SPM12 was used to compare between subjects’ hdEEG signals.

For each subject, the following contrasts were calculated to be used for the analyses of variance (ANOVAs) and correlation analyses: “infant emotional faces versus neutral faces” (D > N, H > N, and A > N).

Brain activity for “infant emotional faces versus infant neutral faces” was then covaried to TAS-20 (F1, F2, and F3) scores, adding the covariate scores to the flexible factorial design of SPM12 one at a time. Positive and negative correlations were assessed. 

The statistical threshold was set at p < 0.01 corrected for multiple comparisons with the family-wise error (FWE_corr_) option at cluster and voxel level, only accepting cluster sizes exceeding 125 voxels. Anatomical regions were identified by Talairach Client 2.4.3 after converting the output isocenter coordinates to Talairach space by using the subroutine implemented by Matthew Brett (http://brainmap.org/index.html).

## 3. Results

### 3.1. Psychological Data

A two-sample T-test revealed significant between-group differences on TAS-20/F3 (Externally-oriented thinking; T_(37)_ = 2.761; *p* = 0.009), and on TAS-20-Tot (T_(37)_ = 2.897; *p* = 0.006), with fathers presenting higher scores both on TAS-20-F3 (M = 19.26; ± 3.68) and TAS-20-Tot (M = 47.21; ± 6.46) than mothers (TAS-20-F3: M = 16.15; ± 3.36; TAS-20-Tot: M = 41.15; ± 6.60). Thus, compared to mothers, fathers in this study reported greater difficulty in emotional self-awareness and a greater tendency to minimize emotional experience by focusing attention externally.

### 3.2. hdEEG Data: Infant Emotional Faces to Neutral Faces

Table 1 shows brain areas in which a signal increase was observed when comparing “infant emotional faces to neutral faces” (D > N, H > N, and A > N), in mothers and fathers, respectively. 

#### 3.2.1. Infant Distressed Faces versus Infant Neutral Faces

When comparing distressed faces to neutral faces (D > N) in mothers, higher activity was found in premotor and primary motor areas (preSMA and precentral gyrus (PrG), both bilaterally), in the right secondary sensorimotor cortex (SPL), and in large areas of the temporal cortex (right inferior temporal gyrus (rITG), medial temporal gyrus (MTG, bilaterally), right medial temporal lobe (rMTL), and left temporal pole (lTP)). 

**Figure 2:**
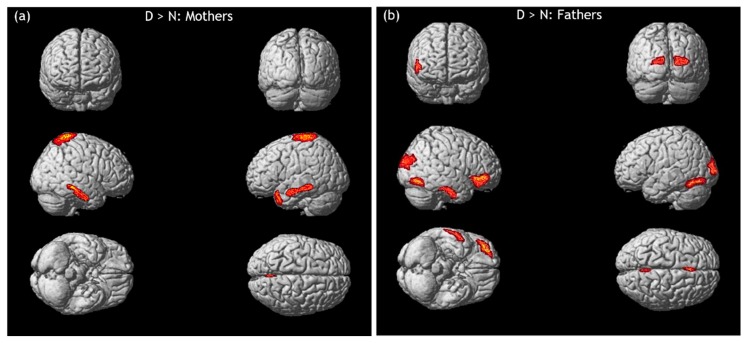
Cortical representation of the cluster of voxels in which the high-density electroencephalography (hdEEG) signal was higher in response to infant distressed emotions (D) than in response to neutral faces (N), in mothers (**a**) and fathers (**b**) respectively. Regional details are presented in Table 1.

In fathers, higher activity was evidenced in right prefrontal regions (IFG and medial frontal gyrus (MFG)), lACC, rSPL, inferior occipital gyrus (IOG, bilaterally), left striate cortex, fusiform gyrus (FuG, bilaterally), and rITG (Figure 2).

#### 3.2.2. Infant Happy Faces versus Infant Neutral Faces

When comparing happy faces to neutral faces (H > N) in mothers, no cerebral signal increase was found. 

In fathers, higher activity was instead evidenced in the left superior and inferior parietal cortex (SPL and angular gyrus (lAG)), in rIOG, and in large regions of the middle, inferior, and superior temporal cortex (rFuG, ITG (bilaterally), and rSTG). 

#### 3.2.3. Infant Ambiguous Faces versus Infant Neutral Faces

When ambiguous faces were compared to neutral faces (A > N) in mothers, higher right activity was observed in DLPFC and MTG. 

In fathers, a signal increase was instead found in large areas of the prefrontal (rIFG, rMFG, FPC (bilaterally), and DLPFC (bilaterally)), parietal (left post-central gyrus (lPoG) and rSPL), occipital (IOG (bilaterally) and right middle occipital gyrus (rmOG)), and temporal cortex (rITG).

### 3.3. Correlations between Psychological Data and hdEEG Data

Correlational analyses were performed between TAS-20 scores (F1, F2, and F3) and brain regions whose activity was enhanced when contrasting “infant emotional faces versus infant neutral faces”.

In mothers, a significant negative correlation was found between TAS-20/F2 scores and the activity in SPL (bilaterally), in response to infant distressed emotions compared to neutral faces (D > N). These results indicated that the more severe a mothers’ difficulties in describing feelings, the lower the bilateral activity in SPL (Table 2).

In fathers, positive correlations were found between scores on TAS-20/F1 and TAS-20/F3 and activity in rITG, in response to distressed (D) and (only for TAS-20/F3) ambiguous (A) and happy faces (H) as compared to neutral faces (N). Significant positive correlations were also found between TAS-20/F1 scores and activity in rIOG and rFuG, as well as between TAS-20/F3 scores and activity in rIOG, in response to happy faces (H) as compared to neutral faces (N). These results indicate that the more severe the difficulties in identifying feelings and the more evident the tendency to minimize emotional experience by focusing on external events, the higher the activity in these cerebral regions (Table 3).

## 4. Discussion

In line with previous investigations on the parental brain, in our study infant emotions elicited, in both mothers and fathers, enhanced cerebral activity in areas of the prefrontal, parietal, and temporal cortex, which are involved in emotion regulation, empathy, and mentalizing (Table 1) [71,72,73,75]. 

Results of our study also seem to indicate that some differences between mothers and fathers are already shaped during pregnancy with the aim of distinguishing—and at the same time maintaining as complementary—maternal and paternal emotional and behavioral contributions in the future relationship with the child. 

The first result, which fully confirmed our original expectations, has been found when parents were confronted to infant distressed emotions (Figure 2). 

In mothers, the emotional resonance (via SPL; that is, the parietal component of MNS) elicited by infant distress (as compared to infant neutral faces) was strictly associated with a “proneness to act”, as indicated by the activation of premotor and motor areas involved in motor planning (pre-SMA) [80] and motor imagery (PrG) [109]. These neural processes were accompanied by the activation of temporal areas involved in the attribution of mental states to others and the theory of mind (MTL, MTG, and TP). TP is a paralimbic area whose activity (in conjunction with the adjacent mPFC) increases when inferring thoughts and feelings of other people are used to guide personal social behaviors [110]. As a part of the hypothalamic-pituitary-adrenal axis (HPA), TP is also involved in emotion processing, since it modulates the amygdala response to stimuli that are perceived as threatening or fearful [110,111]. These motor, emotional, and cognitive functions are fundamental in early attachment bonds because they allow mothers to detect and properly respond to their infants’ cues. Our results seem to show that, in mothers, such processes are already prepared during pregnancy.

Findings on premotor and primary motor activations were not paralleled in fathers, who, in response to infant distressed emotions, showed a signal increase in medial and inferior prefrontal areas (MFG and IFG). The inferior prefrontal cortex is known to also have a key role in interpreting the emotional value of incoming information [112], in integrating multiple sources of information to pursue a higher goal [91,113], as well as allowing empathy (IFG). The paternal empathic response was also reflected by the signal increase found in the sensorimotor component of MNS (SPL). In fathers, infant distress also elicited an enhanced response in ACC, which is known to be involved in emotional awareness and affect regulation. It is worth noting that this cerebral region is also implicated in self-referential processing [114], in emotion-related imagery or recall [115], and in the selection of response between competing thoughts and correcting errors [116]. A further cerebral region, that was highly activated by infant distress in fathers, is ITG. This temporal area is implicated in the attributions of intentions to others [117]. Moreover, in conjunction with the adjacent FuG, ITG is also involved in face perception and recognition as well as in the differentiation of familiar faces from unfamiliar faces, receiving a great contribution from the amygdala, especially during the processing of fearful expressions [118,119,120]. In fathers, enhanced activity was also observed in occipital areas implicated in visual attention (striate and IOG) and (as regards IOG) in visuo-spatial information processing and visual mental imagery [121,122,123]. 

The fact that the same premotor activation in response to infant distressed emotions was not found in fathers may be explained in light of the differential roles mothers and fathers have in the relationship with the child. As Swain et al. have underscored, while empathic/mentalizing neural circuitry implicated in the response to infant cues may be similar for both parents, some differences may be found when taking into account the peculiarities of paternal and maternal contributions in their relationship with children [71]. While in mothers, sensitivity is aimed at restoring a positive emotional balance in the child through the regulation of his/her aversive arousal [12,36], in fathers sensitive behaviors, are instead aimed at encouraging the child to explore the outside world, through vigorous playful stimulations [37,38,39,40].

Consistent with this literature, our findings suggest that, even though infant distress elicited a similar empathic resonance in mothers and fathers, this empathic response was translated into a “promptness to act” only in mothers, maybe because of their higher predisposition to cope with the infant’s negative emotions. 

A significant result, which (only partially) confirm our original hypotheses, was found for infant happy emotions. Contrary to our expectations, when contrasting these emotions to infant neutral faces, no cerebral signal increase was found in mothers. These results seem to show that mothers perceived infant faces as expressing happiness and those expressing emotional neutrality as equally activating. 

This comparison produced very different results in fathers, who showed enhanced activity in parietal (SPL), temporal (ITG and STG), and occipital regions (IOG and FuG) that were fairly similar to those they reported in response to infant distress. Beyond these areas, increased activity was also found in the angular gyrus (AG). It is worth noting that AG is part of a “bottom-up” attentional subsystem that, along with the right IPL, modulates both the automatic allocation and the maintenance of attention to stimuli that are perceived as emotionally salient [124]. These results seem to show that, contrary to mothers, fathers experienced infant happy emotions as more emotionally salient than infant neutral expressions. We may assume that in fathers, this affective orientation towards infant happiness may be already shaped during pregnancy, to prepare them to engage in playful activities with their child after childbirth. This paternal predisposition is very relevant if we consider that the way fathers play with their children plays an important role in fostering children’s socio-emotional development and attachment security [125,126].

A further relevant result was found as regards infant ambiguous emotions.

As predicted, when contrasting infant ambiguous expressions to infant neutral faces, both mothers and fathers showed increased activity in cerebral areas subserving mentalizing processes (MTG for mothers; ITG, FPC, and MFG for fathers) and—contrary to our predictions—in regions implicated in emotional regulation processes (DLPFC) [127]. 

Nevertheless, compared to mothers, fathers also reported increased activity in frontal and parietal regions implicated in empathy (IFG, PoG, and SPL), in temporal and occipital areas involved in visuo-spatial information processing and visual mental imagery (mOG and IOG), as well as in a wider set of areas included in the emotion regulation and executive network, specifically MFG (whose response to infant ambiguous expressions was already found in a previous study [80]) and FPC [91]. It is worth noting that FPC is also known to have a crucial role in the modulation of adults’ emotional response to infant facial emotions [11,19,73,78] and in self-referential and introspectively oriented mental activity [114,128].

Consistent with our expectations, results on ambiguous emotions showed that both mothers and fathers made high efforts to infer the emotional significance of these uncertain facial expressions. Nevertheless, whilst mothers were more inclined to engage in interpreting than in empathizing with ambiguous emotions, fathers were also prone to analyze the perceptual features of infants’ faces, engage in a self-referential activity, and, compared to mothers, activate a broader emotional regulatory response while resonating with infant emotions. We suggest that these results may be interpreted as the expression of the potential significance that these emotions may have for parents. It may be assumed that, compared to fathers, mothers are more apt to try to understand the actual emotional states under uncertain facial expressions of the infants. This attitude may be advantageous for mothers, since it may allow them to reassure the infant if he/she is signaling distress. We believe that further research is needed to clarify how ambiguous infant emotions are processed by parents, during pregnancy.

Results on ambiguous infant emotions, along with those found on infant distress, showed that, differently from mothers, fathers are highly inclined to analyze the perceptual features of infants’ faces and to engage in self-referential processes. We may assume that, in expectant fathers, the high engagement in self-referential processes may be primarily caused by infant faces per se which are perceived as unfamiliar. In line with this, Platek et al. have demonstrated that men use facial resemblance as a self-referent phenotype when asked to make hypothetical parental investment decisions [129,130,131]. It follows that, when confronted with infant emotions, expectant fathers may need more perceptual information about an infant’s face than mothers to familiarize themselves with the observed unknown infant and thus to subjectively feel their emotional states.

From a psychodynamic perspective, the findings of our study may be explained by considering the well-known condition of primary maternal preoccupation [132]. This mindset, which develops during the last months of pregnancy and lasts throughout the first months of her infant’s life, has been described as “almost an illness” that the mother must experience and recover from to provide her infant with an environment that can meet his/her physical and psychological needs. As Leckman et al. have demonstrated, primary preoccupation affects both mothers and, although to a lesser extent, fathers, with the effect of heightening parents’ ability to learn their infant’s emotional cues, anticipate his/her needs, and gradually recognize his/her individuality [133]. 

During pregnancy, because of more intense preoccupations, mothers have the chance to develop mental representations of their infants as well as an empathetic attitude toward them, earlier and faster than fathers. These inclinations will shape the ground in which their child’s sense of security will be rooted [27,28,29,30,31,32]. In mothers, the experience of somatic gestation significantly contributes to increasing the richness and specificity of thoughts about their still unborn infant [134]. Indeed, during pregnancy, maternal representations are sustained by the direct perception of the baby, whose vitality is expressed through movements, body development, and the emotionally salient visual inputs coming from ultrasound images [5,135,136,137]. 

In contrast, expectant fathers make greater efforts than mothers to imagine their infants and their future relationship with them [134]. This is because their emotional relationship with the infant is still indirect, as it is modulated via the mothers’ motivation to share with them emotions and physical perceptions of pregnancy [138]. In line with this, it may be suggested that, compared to what is observed in mothers, in fathers, emotional responsiveness to infants’ distress (or potential distress, as in the case of ambiguous emotions) may develop more gradually, may need greater emotional, cognitive, and perceptual investment, and may fully emerge only when they have the possibility to really interact with their real child [139].

In light of the results of the present study, it may be assumed that differences in maternal and paternal mental constellations during pregnancy [140] may also shape distinct forms of responsiveness towards the emotional facial expressions of unknown infants. 

### Neural Responses to Infant Emotions Vary According to Emotional Self-Awareness, in Mothers and Fathers 

Our investigation revealed significant differences between mothers and fathers on emotional self-awareness. Specifically, compared to mothers, fathers reported a greater difficulty in emotional self-awareness (as indicated by higher TAS-20 total scores) and a greater tendency to minimize emotional experience by focusing attention externally. These results are consistent with those found in previous research, in which men were found to be less aware of their own emotional experience (and thus more alexithymic) than women [141,142,143]. 

Results of our investigation suggest that distinct forms of maternal and paternal responsiveness to infants’ emotions may be affected—already during pregnancy—by parents’ ability to be aware of their own emotional experiences. In our study, we addressed this issue by testing the possible association between parents’ neural responses to infant emotions and parents’ emotional awareness. To do this, correlational analyses were performed between the three subscales of TAS-20 and brain areas whose activity was enhanced when comparing infant emotional faces to neutral faces.

Consistent with our expectations, mothers with lower levels of emotional self-awareness (as defined by higher difficulty describing feelings) showed a lower ability to empathize with the distress of unknown infants (through SPL, that is the sensorimotor representations of those emotions) (Table 2). It may be assumed that expectant mothers who have poor awareness of their own emotional experiences might also have difficulty being emotionally available to attune with and regulate the distress of their own infants after childbirth, and (thus) might not be fully able to emotionally support the harmonic development of their own infants’ socio-communicative competencies [54,55,56,57,59]. If confirmed, these assumptions may have relevant implications in parenting. Indeed, research has provided evidence that the way mothers respond to infant distress during pregnancy is associated with the quality of mother–infant bonding during the postpartum period [25].

In fathers, lower emotional self-awareness (as defined by higher difficulty identifying feelings and a higher tendency to minimize emotional experience by focusing to external events) increased the efforts they made to detect perceptual information about the face of the unknown infants (via IOG, rFuG, and ITG), to use visual mental imagery (via IOG), and to infer infants’ mental states from their overt facial expressions (Table 3). For all we know, to date, this is the first investigation that has addressed the association between emotional self-awareness and paternal responsivity to infant emotions during pregnancy. Therefore, based on our findings and taking into account the existing scientific literature on the postpartum period [54,55,56,57,58], we may assume that expectant fathers with a low awareness of their own feelings might also have difficulty understanding the inner states of their own infants after childbirth, and might need to engage in real interactions to create an emotional bond with them.

## 5. Conclusions

We believe that this study may contribute to expanding the knowledge about the neural response to infant emotions during pregnancy—a period in which parents undergo complex psychological and neurobiological transformations that significantly affect the construction of their parental identity as well as the development of a bond with the still unborn child. Moreover, we hope that, in this study, the inclusion of fathers may provide an important contribution within neuroscientific research on parenting (which, to date, has been mostly focused on their maternal counterparts) through the evidence of the distinct brain patterns that characterize the preparedness towards infant emotional cues during pregnancy in mothers and fathers respectively [19].

To our knowledge, no previous study has ever investigated the relationship between emotional self-awareness and neural response to infant emotions in expectant parents. Overall, results of our study (albeit preliminary) may have great implications for parenting, since they shed light on the support programs that could be designed from the first months of pregnancy to sustain the ability of parents to be in touch with their own emotions and with those of their infants after childbirth. These programs may help parents to more easily respond to infants who are not fully able to send clear social signals, as in the case of pre-term infants [8,9,144]; moreover, they may be particularly relevant for expectant fathers, who are still scantly considered as needing emotional support during pregnancy.

Notwithstanding the reported strengths, our investigation has some notable limitations, the main one being the relatively small number of recruited expectant parents.

We are also aware that we did not consider an important dimension of parenting: that is, the capacity to attribute mental states (such as intentions, motivations, and emotions) to infants. This ability, which has been conceptualized as reflective functioning (RF) [145], is a key component of parental empathy, as shown in previous studies on mothers [80]. We believe that the inclusion of an RF measure (beyond that of emotional self-awareness that we have instead considered) would have provided a clearer and more articulated picture of the possible relationship between psychological dimensions and neural responses to infant emotions, in parents during pregnancy.

Finally, in this study, expectant mothers and fathers were recruited independently from maternity and child health services. This did not allow examination of whether enhanced neural responses to infant emotions in one partner might be (or not be) associated with corresponding increases in the spouse.

Further studies are thus needed, including larger participant groups as well as more articulated psychological measures on mentalizing and emotional self-awareness, to provide a more exhaustive description of the complexity that characterizes the neural processing of infant emotions in expectant mothers and fathers.

## Figures and Tables

**Table 1:** Cerebral regions in which activity was higher in response to infant emotional faces than in response to neutral faces.

CONTRAST	MOTHERS							FATHERS						
	X	Y	z	T	Cluster	Region	BA	x	Y	Z	T	Cluster	Region	BA
D > N	16	−24	68	10.28	610	r preSMA	6	-	-	-	-	-	-	-
	−4	−34	70	9.16	688	l preSMA	6	-	-	-	-	-	-	-
	−14	−26	68	9.60	688	l PrG	4	-	-	-	-	-	-	-
	6	−40	70	14.74	610	r PrG	4	-	-	-	-	-	-	-
	-	-	-	-	-	-	-	40	38	0	13.49	811	r IFG	47
	-	-	-	-	-	-	-	48	30	12	13.20	811	r MFG	46
	-	-	-	-	-	-	-	0	6	20	8.96	568	l ACC	33
	10	−72	54	6.24	610	r SPL	7	34	−66	46	8.31	104	r SPL	7
	-	-	-	-	-	-	-	24	−90	2	12.69	352	r IOG	19
	-	-	-	-	-	-	-	−30	−84	8	7.10	173	l IOG	19
	-	-	-	-	-	-	-	−18	−94	4	8.07	173	l Striate	17
	-	-	-	-	-	-	-	38	−48	−20	16.02	346	r FuG	37
	-	-	-	-	-	-	-	−36	−52	−20	16.15	323	l FuG	37
	58	−38	−26	8.11	155	r ITG	20	48	−12	−38	7.32	290	r ITG	20
	64	−16	−18	9.07	155	r MTG	21	-	-	-	-	-	-	-
	−50	4	−34	9.67	106	l MTG	21	-	-	-	-	-	-	-
	14	−34	−4	13.03	294	r MTL	27							
	−46	12	−32	7.97	106	l TP	38	-	-	-	-	-	-	-
H > N	-	-	-	-	-	-	-	−30	−68	50	10.41	138	l SPL	7
	-	-	-	-	-	-	-	−54	−62	8	11.00	145	l AG	39
	-	-	-	-	-	-	-	48	−76	−4	8.61	234	r IOG	19
	-	-	-	-	-	-	-	40	−66	0	8.89	234	r FuG	37
	-	-	-	-	-	-	-	46	−18	−28	11.94	456	r ITG	20
	-	-	-	-	-	-	-	−44	−14	−32	11.28	251	l ITG	20
	-	-	-	-	-	-	-	52	−62	14	11.42	136	r STG	22
A > N	-	-	-	-	-	-	-	40	40	−4	13.65	890	r IFG	47
	-	-	-	-	-	-	-	38	34	8	13.89	890	r MFG	46
	-	-	-	-	-	-	-	38	46	10	8.35	890	r FPC	10
	-	-	-	-	-	-	-	−16	54	26	8.40	237	l FPC	10
	40	16	42	12.83	165	r DLPFC	9	16	52	24	8.36	208	r DLPFC	9
	-	-	-	-	-	-	-	−6	52	30	7.31	237	l DLPFC	9
	-	-	-	-	-	-	-	−46	−26	58	15.06	149	l PoG	3
	-	-	-	-	-	-	-	38	−68	46	6.91	121	r SPL	7
	-	-	-	-	-	-	-	32	−84	6	13.02	335	r IOG	19
	-	-	-	-	-	-	-	−34	−76	12	14.05	371	l IOG	19
	-	-	-	-	-	-	-	18	−100	8	7.57	335	r mOG	18
	-	-	-	-	-	-	-	48	−12	−38	7.84	365	r ITG	20
	52	8	−32	7.60	198	r MTG	21	-	-	-	-	-	-	-

*Note:* D = Distress; A = Ambiguous emotions; H = Happy emotions; N = Neutral expressions. Only peaks of clusters corrected for multiple comparison at cluster level p < 0.001 are reported. BA = Brodmann Area; ACC = Anterior Cingulate Cortex; AG = Angular Gyrus; Frontopolar Cortex; FuG = Fusiform Gyrus; IFG = Inferior Frontal Gyrus; IOG = Inferior Occipital Gyrus; IPL = Inferior Parietal Lobule; ITG = Inferior Temporal Gyrus; MFG = Medial Frontal Gyrus; mOG = Middle Occipital Gyrus; MTG = Medial Temporal Gyrus; MTL = Medial Temporal Lobe; PoG = Postcentral Gyrus; pre−SMA = pre-Supplementary Motor Area; PrG = Precentral Gyrus; SPL = Superior Parietal Lobule; STG = Superior Temporal Gyrus; Striate = Striate Cortex; TP = Temporal Pole; r = right; l = left.

**Table 2:** Brain regions whose activity was negatively correlated to TAS-20 scores in mothers when contrasting infant emotional faces to infant neutral faces.

Clinical Scales		Contrast	X	Y	z	T	Cluster	Region	BA
TAS-20	F2	D > N	10	−72	54	6.24	239	r SPL	7
			−12	−70	54	7.23	370	l SPL	7

Note: F2 = Difficulty describing feelings. D = Distress; N = Neutral expressions. BA = Brodmann Area; SPL = Superior Parietal Lobule; r = right; l = left.

**Table 3:** Brain regions whose activity was positively correlated to TAS-20 scores in fathers, when contrasting infant emotional faces to infant neutral faces.

Clinical Scales		Contrast	X	Y	Z	T	Cluster	Region	BA
TAS−20	F1	D > N	48	−12	−36	7.20	133	r ITG	20
		H > N	42	−72	−8	8.35	133	r IOG	19
			40	−66	0	8.89	142	r FuG	37
	F3	D > N	48	−12	−38	7.32	269	r ITG	20
		H > N	42	−72	−8	8.35	111	r IOG	19
			46	−16	−34	10.55	153	r ITG	20
		A > N	48	−12	−38	7.84	306	r ITG	20

*Note:* F1 = Difficulty identifying feelings; F3 = Externally oriented thinking. D = Distress; A = Ambiguous emotions; H = Happy emotions; N = Neutral expressions. BA = Brodmann Area; FuG = Fusiform Gyrus; IOG = Inferior Occipital Gyrus; ITG = Inferior Temporal Gyrus.
